# Coastal and deep-sea biodegradation of polyhydroxyalkanoate microbeads

**DOI:** 10.1038/s41598-024-60949-z

**Published:** 2024-05-05

**Authors:** Natsumi Hyodo, Hongyi Gan, Manikandan Ilangovan, Satoshi Kimura, Ken-ichi Kasuya, Noriyuki Isobe, Tadahisa Iwata

**Affiliations:** 1 Science of Polymeric Materials, Department of Biomaterial Sciences, Graduate School of Agricultural and Life Sciences, The University of Tokyo, 1-1-1 Yayoi, Bunkyo-Ku, Tokyo, 113-8657 Japan; 2 Green Polymer Research Laboratory, Graduate School of Science and Technology, Gunma University, Kiryu, Gunma 376-8515 Japan; 3 Biogeochemistry Research Center, Research Institute for Marine Resources Utilization (MRU), Japan Agency for Marine-Earth Science and Technology (JAMSTEC), 2-15 Natsushima-Cho, Yokosuka, Kanagawa 237-0061 Japan

**Keywords:** Environmental sciences, Polymers

## Abstract

Microbeads find widespread usage in personal care items and cosmetics, serving as exfoliants or scrubbing agents. Their micro-scale size poses challenges in effective drainage capture and given their origin from non-biodegradable oil-based plastics, this contributes substantially to marine pollution. In this study, microbeads were prepared by a simple yet scalable melt homogenization method using four types of polyhydroxyalkanoates (PHA), namely poly[(*R*)-3-hydroxybutyrate] (P(3HB)), poly[(*R*)-3-hydroxybutyrate*-co-*(*R*)-3-hydroxyvalerate] (P(3HB-*co*-3HV)), poly[(*R*)-3-hydroxybutyrate-*co*-(*R*)-3-hydroxyhexanoate] (P(3HB-*co*-3HHx)) and poly[(*R*)-3-hydroxybutyrate-*co*-(*R*)-4-hydroxyvalerate] (P(3HB-*co*-4HB)). Microbeads with different surface smoothness, compressive strength (6.2–13.3 MPa) and diameter (from 1 ~ 150 μm) could be produced. The microbeads were subjected to a comprehensive degradation analysis using three techniques: enzymatic, Biochemical Oxygen Demand (BOD) evaluations, and in situ degradation tests in the deep-sea off Misaki Port in the northern Pacific Ocean (depth of 757 m). Qualitatively, results from enzymatic and in situ degradation demonstrated significant degradation within one week and five months, respectively. Quantitatively, BOD findings indicated that all PHA microbeads degraded similarly to cellulose (~ 85% biodegradability in 25 days). In conclusion, PHA microbeads from this study exhibit promising potential as alternatives to conventional non-biodegradable microbeads.

## Introduction

Petroleum-based plastics are used in various applications as lightweight, inexpensive, and easily moldable materials, and they are indispensable in our daily lives. However, in recent years, marine pollution caused by plastics leaking into the environment has become a pressing issue^1,2^. In particular, the adverse effects of microplastics have gained significant attention. Microplastics are defined as plastic particles with a diameter of less than 5 mm^3^. Plastic particles intentionally created in small sizes are classified as “primary microplastics”, while those reduced in size through the weathering of larger plastics are classified as “secondary microplastics”^4,5^. These microplastics drift in the ocean for extended periods, and when ingested by marine organisms, cause adverse physical effects such as damage to the digestive organs^6,7^. Moreover, these microplastics can absorb environmental hormones and other toxic substances, which can enter and bioaccumulate across different levels of the ecosystem^8–11^.

Microbeads are a type of primary microplastic with diameter less than 1 mm^12^. They are commonly found in personal care products, such as face washes and toothpaste, and have been primarily used as scrubs to remove dirt and dead skin cells^13^. Besides their role as scrubs, they are also used in cosmetics due to their excellent texture and optical properties^14^. Conventional microbeads are typically made from non-biodegradable petroleum-based synthetic plastics like polyethylene (PE), polypropylene (PP), polystyrene (PS). These microbeads cannot be removed by sewage treatment, leading to an increase in marine pollution^15^. Consequently, many countries are imposing restrictions or bans on the use of these non-biodegradable microbeads^14,16^. Hence, there is a growing need to develop alternatives that can biodegrade when released into the environment.

**Figure 1:**
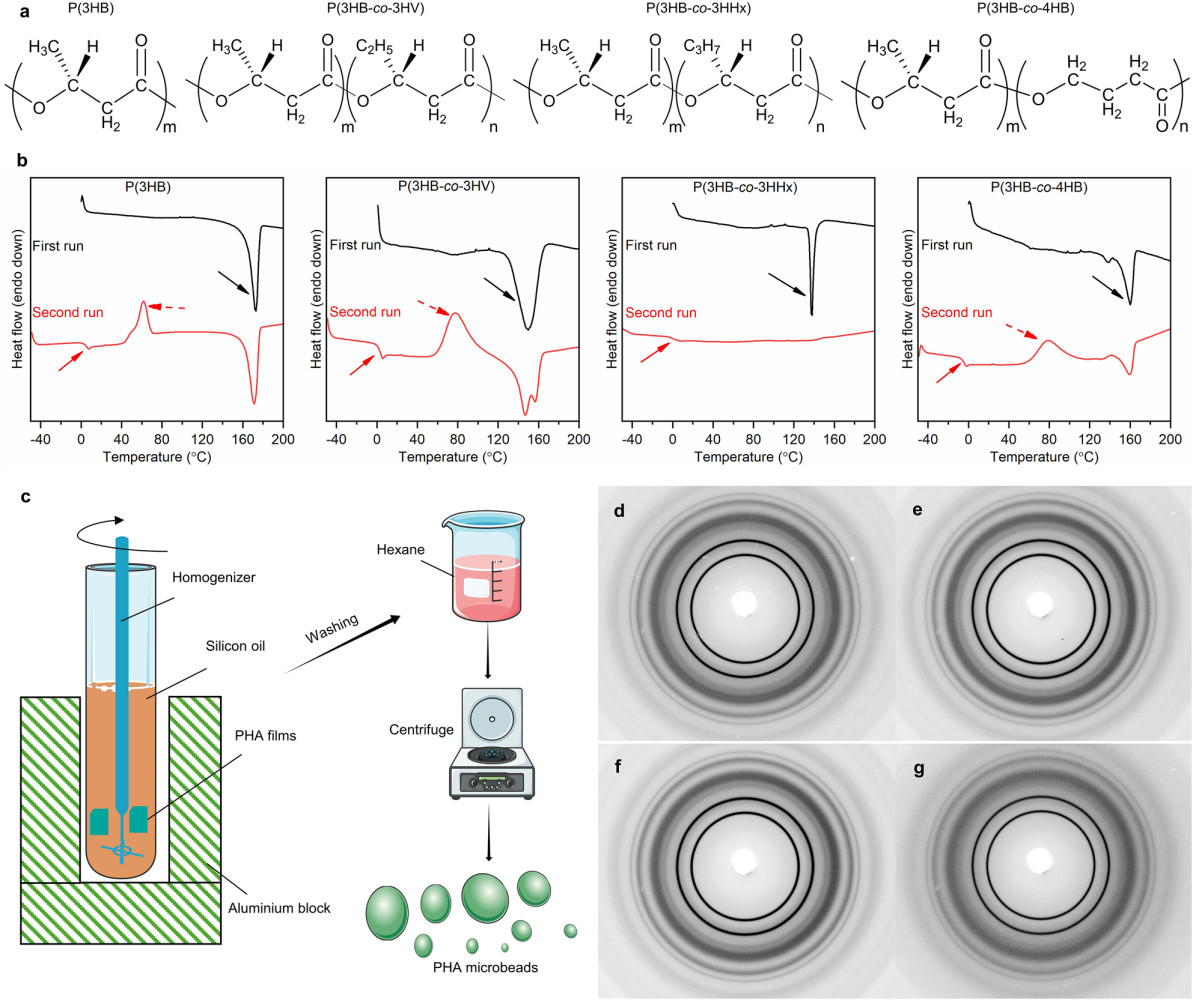
Processing, thermal and crystal properties of PHA microbeads. (**a**) Chemical structure of P(3HB), PHBV, PHBH and PHB4HB. (**b**) First and second run DSC curve of PHA neat samples. The arrows in the first run indicate the T_m_. The solid and dotted arrows in the second run indicate T_g_ and T_c_ respectively. (**c**) Fabrication scheme of PHA microbeads (**d**–**g**) 2D WAXD images of P(3HB), PHBV, PHBH and PHB4HB respectively. Refer Table 1 for the summary of thermal properties and crystallinity.

Therefore, the focus of attention is polyhydroxyalkanoate (PHA), a polymer produced by microbial biosynthesis from biomass such as sugars and vegetable oils. PHA has good thermoplasticity, and excellent biodegradability in various environments, including the deep ocean^17–22^. Poly[(*R*)-3-hydroxybutyrate] (P(3HB)), a typical PHA polymer, is a thermoplastic with a high melting point (T_m_: ~ 175 °C) but is known to be a hard and brittle material. Furthermore, it has the disadvantage of secondary crystallization at room temperature, which can cause degradation over time^23,24^. However, the disadvantages of P(3HB) can be improved by introducing a second component into P(3HB) to make a copolymer. Typical examples of copolymers are poly[(*R*)-3-hydroxybutyrate-*co*-(*R*)-3-hydroxyvalerate] (P(3HB-*co*-3HV))^25^, poly[(*R*)-3-hydroxybutyrate-*co*-(*R*)-3-hydroxyhexanoate] (P(3HB-*co*-3HHx))^26,27^, poly[(*R*)-3-hydroxybutyrate-*co*-(*R*)-4-hydroxyvalerate] (P(3HB-*co*-4HB))^28,29^ (Fig. 1a). P(3HB-*co*-3HV) exhibits stiffness and strength, P(3HB-*co*-3HHx) is flexible, and P(3HB-*co*-4HB) offers flexibility and stretchability. PHA holds great potential as a biodegradable polymer with excellent applicability owing to its tunable properties by copolymerization.

Microbeads are generally prepared by methods such as emulsification, spray-drying and ionic gelation, and there have been several reports of biodegradable microbeads produced by these methods^30–32^. However, these processes often involve the use of toxic solvents such as chloroform and dichloromethane for polymer dissolution, which on a sustainability point of view is often viewed as a limitation.

In this study, we aimed to prepare PHA microbeads using the melt homogenization method, a more environmentally friendly and scalable process that eliminates the need for hazardous organic solvents. Our work utilizes a straightforward approach leveraging one of the most advantageous properties of thermoplastics, their ability to be heat-processed. For the preparation, four types of PHA were used: P(3HB) and its copolymers, including P(3HB-*co*-8 mol%-3HV) (PHBV), P(3HB-*co*-6 mol%-3HHx) (PHBH), P(3HB-*co*-8.9 mol%-4HB) (PHB4HB). The morphology, compressive strength, and biodegradability of each microbeads were subsequently evaluated to assess its potential as ecofriendly bio-based alternatives to conventional petroleum-derived microbeads. Especially with regard to biodegradability, three different techniques are investigated: (1) enzymatic degradation, (2) biochemical oxygen demand (BOD) using seawater from the Tokyo Bay, and (3) deep-sea degradation at off Misaki Port (depth 757 m), in the northern Pacific Ocean, carried out with the help of human occupied *Shinkai6500* submersible.

## Results and discussion

### Preparation and characterization of the microbeads

The basic material properties of PHA neat and microbeads samples are shown in Table 1. The processing temperature of each sample was determined to be 190 °C for P(3HB) and 180 °C for PHBV, PHBH, and PHB4HB, exceeding the obtained T_m_ (Fig. 1b). A simple schematic of the microbeads fabrication is shown in Fig. 1c. It has been reported that the molecular weight of PHA decreases with time when held isothermally at temperatures above the melting point, and that the rate of decrease in molecular weight tends to be greater at higher holding temperatures. As expected, after melt-processing, the M_w_ of P(3HB) decreased from 600,000 to 220,000, whereas the M_w_ of PHBV decreased from 590,000 to 320,000 when formed into microbeads. On the other hand, PHBH and PHB4HB showed almost no change in M_w_ at the powder or microbeads stage. This could be attributed to the higher activation energy of PHBH and PHB4HB leading to better thermal resistance^33–35^.

**Table 1:** Summary of thermal properties, molecular weight, and crystallinity of PHA powder and microbeads.

Sample		DSC^1^			GPC^2^		WAXD
		T_g_ (°C)	T_c_ (°C)	T_m_ (°C)	M_w_ × 10^4^	M_w_/M_n_	Crystallinity (%)
P(3HB)	Powder	4	62	171	60	1.97	
	Microbeads				22	2.30	65
PHBV	Powder	2	77	147,157	59	2.81	
	Microbeads				32	2.17	64
PHBH	Powder	1	–	138	29	2.12	
	Microbeads				26	2.25	52
PHB4HB	Powder	− 6	79	160	4.3	3.11	
	Microbeads				4.0	2.80	50

^1^T_m_ was determined from the first heating run, Tc from cooling curve and T_g_ from the second heating run.

^2^Molecular weight was determined after dissolving the samples in CHCl_3_.

Two dimensional WAXD images of the synthesized microbeads are shown in Fig. 1d-g. The crystallinity of P(3HB) and PHBV microbeads were approximately 65%, while that of PHBH and PHB4HB microbeads were comparatively lower at 50% (Table 1). The higher crystallinity in PHBV is due to the incorporation of 3HV units into the crystals of the 3HB chain^36–38^. In contrast, the 3HHx and 4HB units are not included in the crystal structure of the 3HB chain portion, reducing the overall crystallinity of PHBH and PHB4HB^39,40^.

**Figure 2:**
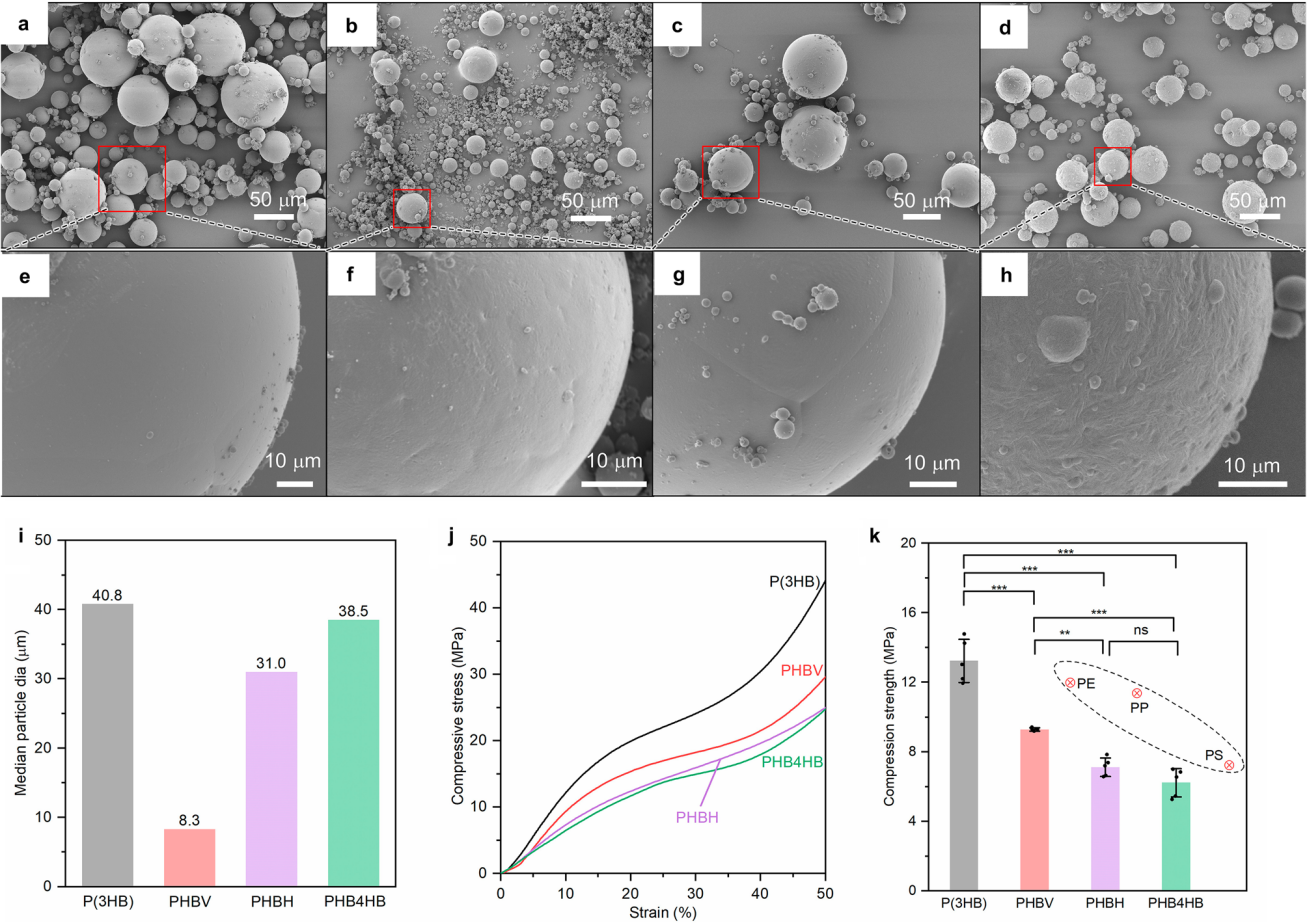
Morphological characteristics and compressive strength of PHA microbeads. (**a**–**h**) Shows the SEM images of P(3HB) (**a**,**e**), PHBV (**b**,**f**), PHBH (**c**,**g**), PHB4HB beads (**d**,**h**). The images shown are representative of n = 3 samples. The morphology remained consistent irrespective of fabrication lots. (**i**) Median particle size of the prepared PHA microbeads. (**j**) Stress–strain curve of the PHA microbeads obtained during the compression test. The graph represents the stress–strain profile of the sample closest to the average value. (**k**) Compressive strength of P(3HB), PHBV, PHBH and PHB4HB microbeads at 10% strain. The value shown here is the average of n = 5 distinct samples picked randomly from different preparation lots. The error bar represents the standard deviation. The results were compared using a one-way ANOVA with post-hoc Tukey test. **p < 0.05, ***p < 0.01, *ns* not significant. The red marker represents the compressive strength of conventional microbeads^45^.

SEM images of the synthesized PHA microbeads are presented in Fig. 2a–h. P(3HB), PHBV, and PHBH microbeads were spherical with a smooth surface (Fig. 2a–c,e–g). In contrast, PHB4HB microbeads were also spherical but had rough surfaces (Fig. 2d,h). This difference in surface morphology may be attributed to the rubber-like elasticity of PHB4HB with ~ 40 mol% 4HB^41,42^, and variations in viscosity of the PHA film when melted in silicone oil. Particle size distribution of each PHA microbead showed that more than half of the PHA microbeads had a particle size of 50 μm or smaller, however it was also observed that there were beads with greater sizes (Fig. S1). Therefore, by sorting out microbeads with uniform particle sizes through classification, it is possible to use microbeads of various sizes for specific applications. The pore diameter of human skin is reported to be 250–500 μm^43^. Since the microbeads produced in this study are smaller (Fig. 2i) than the pore diameter, they are expected to be used in scrubbing agents for penetrating into pores to remove dirt. Additionally, they can be utilized in cosmetics to reduce skin surface unevenness and make pores less noticeable.

The mechanical properties of microbeads were evaluated by compression testing (Fig. 2j,k). Figure 2j shows the stress–strain curves of microbeads with particle sizes less than 50 μm. Since the microbeads were plastically deformed and did not fracture during compression, the stress at 10% strain was used as the compressive strength. The P(3HB) microbeads exhibited the highest compressive strength of 13.3 MPa, while the PHBV, PHBH, and PHB4HB microbeads exhibited 9.3 MPa, 7.4 MPa, and 6.2 MPa, respectively, (Fig. 2k) indicating a change from hard to soft properties. This change may be attributed to the hard and brittle nature of P(3HB), whereas the other PHAs exhibit soft properties due to the reduction in crystallinity caused by the introduction of the copolymer component. In particular, PHBH and PHB4HB microbeads have lower crystallinity than P(3HB) microbeads, as discussed earlier suggesting a significant effect on compressive strength. Toyoda et al. reported that the mechanical properties of microbeads correlate with the feel properties such as "smoothness" and "moistness"^44^, suggesting that PHA microbeads can provide a wide range of feel properties. Comparing the compressive strength of PHA microbeads and conventional petroleum-derived microbeads^45^, P(3HB) showed higher strength than PE (11.8 MPa) and PP (11.2 MPa), while PHBV showed slightly lower strength. PHBH and PHB4HB were confirmed to have similar strength to PS (6.8 MPa). As discussed earlier, the M_w_ of P(3HB) and PHBV decreased when formed into microbeads, but this decrease in M_w_ is not particularly problematic since the compressive strength is comparable to that of petroleum-derived microbeads. Based on these results, PHA microbeads can be expected to be used as an alternative to petroleum-derived microbeads in a wide variety of applications.

### Enzymatic degradation tests on the PHA microbeads

**Figure 3:**
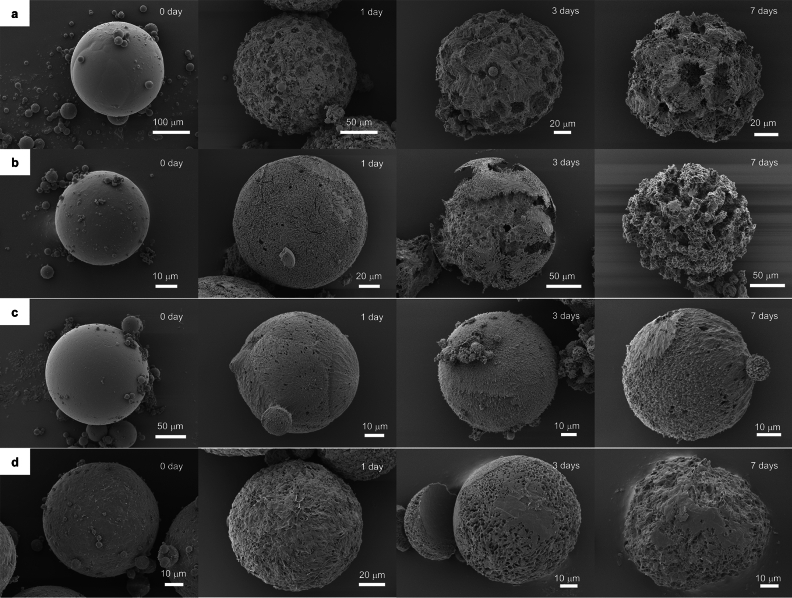
Morphological evolution of PHA microbeads during the course of enzymatic degradation. Each row depicts the morphology of microbeads from different PHA. (**a**) Morphological characteristics of P(3HB) microbeads before and after 1-, 3- and 7-days of enzymatic degradation (**b**–**d**) indicate the surface morphology of PHBV, PHBH, and PHB4HB, respectively. The images are representative of triplicate samples conducted simultaneously. The buffer and aqueous solution of the enzymes were replaced constantly to ensure the activity of the enzymes remain unchanged.

The morphological changes of PHA microbeads were observed by SEM after 1, 3, and 7 days of enzyme addition (Fig. 3). All microbeads exhibited increased surface roughness one day after enzyme addition, confirming the initiation of degradation. P(3HB) and PHBV microbeads showed progressive internal degradation after the third day. In contrast, PHBH and PHB4HB microbeads exhibited gradual surface-to-interior degradation. The observed differences in the degradation morphology are believed to be influenced by the crystallinity variations, depending on whether the crystalline part contains a secondary component. In the case of PHBV, the 3HV units of the second component are incorporated into the crystalline region of the 3HB chain, so P(3HB) and PHBV microbeads contain many large crystals, as evident from the observed spherulites in Fig. 3a,b. Consequently, it is presumed that the degradation enzyme has penetrated internally at an early stage, as preferential degradation occurred from the amorphous part around the crystals. On the other hand, PHBH and PHB4HB exclude the 3HHx and 4HB units of the second component from the crystalline region of the 3HB chain. Therefore, the crystal size of PHBH and PHB4HB microbeads is small, and both the crystalline and amorphous parts are degraded at a similar rate, suggesting that degradation gradually progresses from the surface of the microbeads.

### Biodegradability of PHA microbeads in deep-sea floor

**Figure 4:**
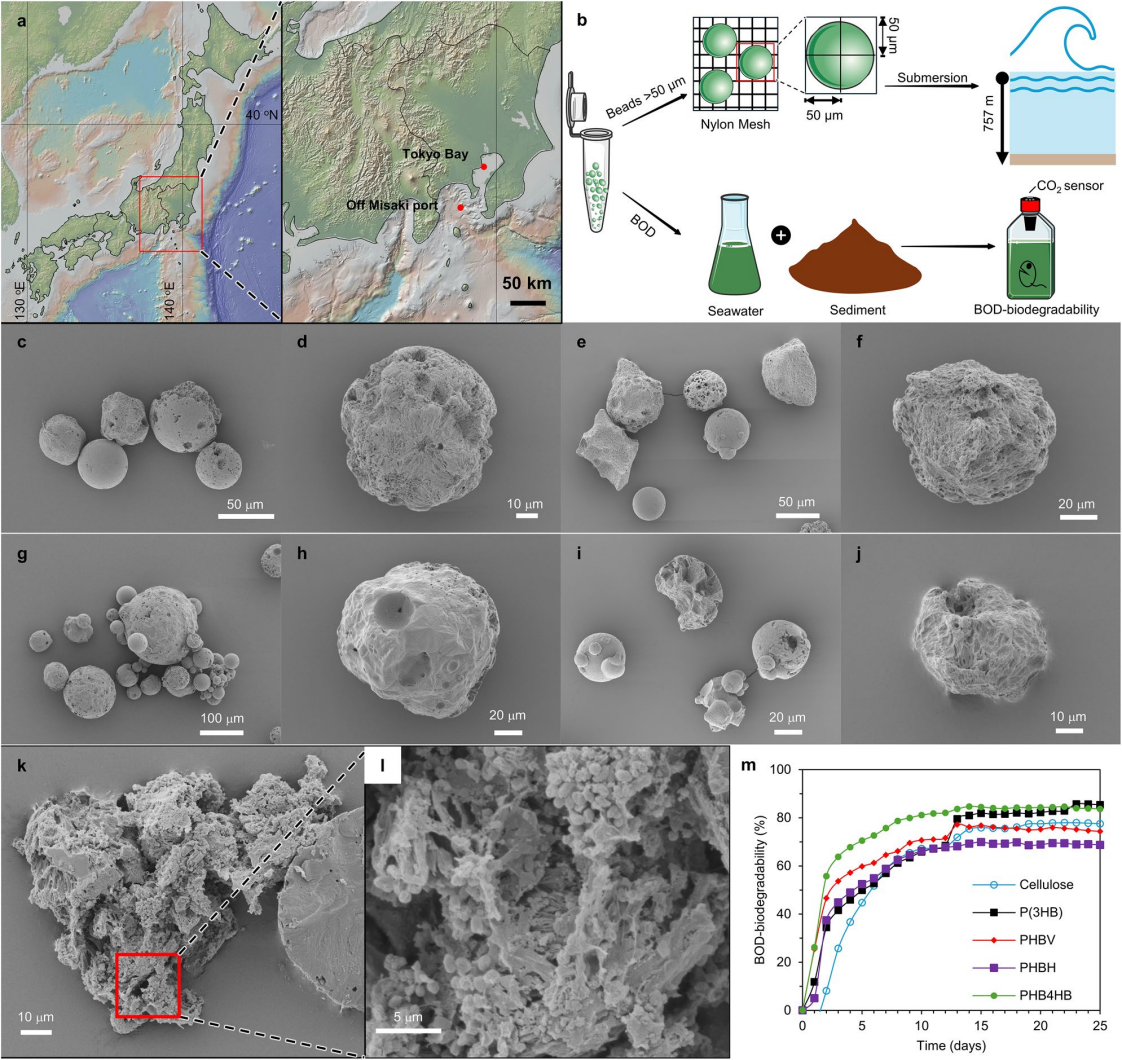
Marine degradation of PHA microbeads carried out under real-life conditions. (**a**) Map of Japan indicating the locations of in situ degradation test site (Bathyal seafloor off Misaki port) and seawater collection spot for BOD tests (near the Tokyo Bay). The map was created using GeoMapApp (geomapp.org) / CC BY (Ryan et al.^53^). (**b**) Schematic representation of the environmental degradation tests of PHA microbeads. The submersion and recovery were carried out using the human operated *Shinkai6500* submersible aboard its support vessel *RV Yokosuka*. (**c**–**j**) SEM images of microbeads after 5 months of submersion in the deep-sea floor. (**c**,**d** P(3HB), **e**,**f** PHBV, **g**,**h** PHBH, i,j PHB4HB microbeads). The images are representative of n = 3 samples. (**k**,**l**) Surface morphology of PHB4HB after fixing microorganisms using formaldehyde solution. (**m**) BOD-biodegradability curve of PHA microbeads and cellulose standard using seawater from Tokyo bay. The curves are representative of n = 3 samples. Refer Table 2 for more details on the environmental degradation sites.

Since PHA microbeads have a greater density than water, those that enter the ocean may sink to the deep sea. Therefore, an in situ deep-sea degradation test of PHA microbeads was conducted at a depth of 757 m off Misaki Port (Fig. 4a, Table 2). Initially, all the PHA microbeads prepared in this study, with a particle size of  50 µm or larger, were enclosed in nylon mesh bags featuring a 50 µm mesh opening (Fig. 4b). These bags were then submerged for a period of 5 months and was subsequently recovered and characterized.

**Table 2:** Geographical, environmental and experimental details of in situ degradation and BOD tests.

Sampling Site	Latitude	Longitude	Water temperature (°C)	pH	Sampling time	Other information
Bathyal seafloor off Misaki port	35°4′ N	139°32′ E	4.4	7.9	Submerged: Jun. 6, 2023 Recovered: Nov. 3, 2023	Dissolved oxygen: 2.1 mg/L
Tokyo Bay	35°37′ N	139°46′ E	27	7.6	Seawater collected: Sept. 8, 2022	Viable microorganism counts: 8.9 × 10^3^ CFU/mL

As shown in Fig. 2a–h, the surface of the neat PHA microbeads was smooth, whereas almost all the microbeads after being placed in deep sea showed small holes and irregularities on the surface (Fig. 4c–l) indicating gradual progression of degradation. Some of the beads have also lost its sphericity, turning into wedge shaped particles (Fig. 4e,i,j). Appearance of spherulite-like structures can also be confirmed in the microbeads after submersion in deep sea (Fig. 4d,f). To analyze the attached microbes, PHB4HB microbeads were immobilized in formaldehyde and observed in SEM (Fig. 4k,l). Morphology of those beads revealed the formation of a uniform aggregate structure comprised of microbeads and biofilm with a large number of *cocci* (size ~ 1–2 µm) attached to the surface. This is contrary to past reports in microplastics with smooth surface morphology, where biofilms were mainly concentrated in the wrinkles of the particles^46^. Although biofilm formation has been confirmed in PHA films^22^, no clear reports have yet been published on PHA based microbeads. The results from this study indicate that microbes are able to form biofilms on microbeads as well. In fact, this is quite surprising that biofilms can form in particles with dia 50–200 µm, as typical bacteria itself is only on average ~ 2 µm in size^47^. However, unlike in PHA film or fiber samples, we could not confirm the presence of any rod-like bacilli on the surface^48^. This could be potentially due to the smaller size of the microbeads compared to films/fibers making it difficult to attach on the surface. Nevertheless, on comparing the morphology of the in situ degradation samples with that of the enzymatically degraded microbeads (Fig. 3), we can see a similar pattern overall which indicates that the PHA microbeads degraded through the action of the extracellular enzymes secreted by the microbes in the biofilm. Since the nylon mesh used in the study had a gap of 50 µm, particles that degraded and reduced to < 50 µm might have escaped the mesh and thus it is difficult to quantify the weight loss of PHA microbeads. Having said that, the recovery rate by weight% of the beads after submersion in deep sea were 45 wt% for P(3HB), 44% for PHBV, 20% for PHBH and 52% for PHB4HB. Meanwhile, the BOD degradability of PHA microbeads (discussed later) shows near complete degradation which suggests that the beads that escaped the mesh would also eventually degrade. Overall, these results indicate that biodegradation of PHA microbeads proceeds even when they are discharged into the ocean and submerged in deep water, potentially reducing the environmental damage caused by the runoff microbeads.

### BOD biodegradability of PHA microbeads in seawater

BOD biodegradability tests were conducted using seawater from Tokyo Bay (Fig. 4a,b, Table 2) to quantitatively assess the degradation of PHA microbeads. Figure 4m illustrates the BOD biodegradability results of P(3HB), PHBV, PHBH, PHB4HB microbeads, and cellulose as the reference. After 25 days, the BOD biodegradability was in the order P(3HB) 85% > PHB4HB 83% > Cellulose 77% > PHBV 74% > PHBH 68%. All the test samples showed a very steep initial BOD-degradability rate of 35–55% indicating a faster conversion rate of PHA into CO_2_ and H_2_O by the microorganism in the seawater. In fact, the BOD-degradability of PHA in Day 2 was almost 3.2 to 5.8 × faster than that of cellulose powder. Prior studies have shown that there is a delay, often up to 10 days, between the start of test and initiation of degradation in PHA films and powder mainly attributed to the geometry of the test sample^49^. From the results of successful biofilm formation on PHA microbeads and the steep BOD-curve in this study, it can be said that the formation of biofilms could have been accelerated due to the large surface area of the microbeads leading to faster initiation of degradation. The final BOD biodegradability of all samples saturated at around 75–85% without reaching 100%, likely because some of the low-molecular-weight compounds are used for biomass formation in the microbial cells^50^. The detailed effects of bead size and crystallinity on the rate and extent of environmental degradability of PHA microbeads remains to be analyzed. Overall, PHA microbeads hold promise as an eco-friendly alternative to conventional non-biodegradable microbeads, especially in terms of marine degradability.

## Conclusions

In this study, microbeads were successfully prepared from four types of PHA: P(3HB), PHBV, PHBH, and PHB4HB, by melt homogenization method. All the microbeads obtained were uniformly spherical, with sizes ranging widely from about 1 μm to 150 μm. The compressive strength of the PHA microbeads demonstrated values that broadly covered the strength of conventional petroleum-derived microbeads, such as PE, providing a versatile range of strengths from rigid to flexible. In addition, it was confirmed that PHA microbeads showed high degradability in marine environments, and the biodegradation progressed even in the deep sea. Therefore, the PHA microbeads produced in this study hold promise as sustainable alternative to conventional microbeads, not only in terms of physical property but also in terms of biodegradability.

## Methods

### Materials

P(3HB) and P(3HB-*co*-8 mol%-3HV) were purchased from Imperial Chemical Industries (UK), P(3HB-*co*-6 mol%-3HHx) from Kaneka (Japan), and P(3HB-*co*-8.9 mol%-4HB) from Mirel (USA). Hexane, ethanol were obtained from FujiFilm Wako Pure Chemicals (Japan), and silicone oil from Shin-Etsu Chemical Co., Ltd. (Japan). All the chemicals and reagents were used as received without any further purification.

### Differential scanning calorimetry (DSC)

The thermal properties of each PHA sample were determined using differential scanning calorimetry (DSC8500, Perkin Elmer, USA) under a nitrogen atmosphere. The measurements were performed by sealing 2–3 mg of each sample in aluminum pans. In the first heating scan, the sample was held at 0 °C for 3 min, followed by heating to 200 °C at a heating rate of 20 °C /min. Subsequently, a rapid quench to − 50 °C at 200 °C /min was carried out, and the sample was held at − 50 °C for 3 min. Finally, a second heating scan was conducted at a rate of 20 °C/min from − 50 °C to 200 °C.

### Preparation of PHA melt-pressed films

PHA powders were processed into melt-pressed films to address issues such as poor settling in silicone oil and adherence to test tubes during the preparation of microbeads. The PHA melt-pressed films were prepared using a table-top melt-press (Mini Test Press, Toyo Seiki, Japan). The processing temperatures were determined based on the results of Differential scanning calorimetry (DSC) (Enzymatic degradation tests on the PHA microbeads) and set above the respective melting points (T_m_). For P(3HB), the temperature was set to 190 °C, and for all the other PHAs, to 180 °C. Approximately 1.5 g of each PHA powder was melt-pressed at 5 MPa for 1 min to achieve a thickness of about 300 µm. After pressing, the samples were cooled in ambient air.

### Preparation of microbeads

First, 20 mL of silicone oil was poured into a test tube and using an aluminum block thermostatic bath (DTU-1C; TAITEC, Japan), each sample was heated to its processing temperature. To this heated oil, about 1.5 g of finely cut, melt-pressed films were added and melted for 10 min. The resulting solution was then homogenized using a homogenizer (Microtec, Japan) equipped with a generator shaft (NS-10; 10.5f × 140 mm; Microtec, Japan). The stirring speed was set at 25,000 rpm, and the process lasted for 90 s. Finally, the test tubes were allowed to stand at room temperature until they cooled. The microbeads obtained in the silicone oil were washed with hexane and separated using a centrifuge machine (MDX-310; TOMY SEIKO, Japan) at 360×*g*, 25 °C. The washing process was repeated four times, and the microbeads were air-dried for one day.

### Morphological analysis

The morphology of the prepared microbeads was observed using a scanning electron microscope (JCM-7000; JEOL, Japan) after coating the surface with gold using a magnetron sputter (MSP-1S; VACUUM DEVICE, Japan). The acceleration voltage during observation was set to 5 kV.

### Molecular weight

The number- and weight-average molecular weight (M_n_ and M_w_), and the polydispersity values (M_w_/M_n_) of each sample were evaluated using a GPC system (CBM-20A, RID-20A; SHIMADZU, Japan) in chloroform at 40 °C. Shodex columns (K-806M, K-802) were used at a flow rate of 0.8 mL/min, and a calibration curve was constructed using polystyrene (PS) standards (Shodex).

### Wide-angle X-ray diffraction (WAXD)

The crystallinity of PHA microbeads was determined by WAXD measurements using an X-ray generator (MicroMax-007HF, Rigaku, Japan) at 40 kV and 30 mA, and wavelength of 0.15418 nm (Cu-Kα radiation). The camera length was calibrated using Si powder and set to 54.5 mm. PHA microbeads were sealed in a 1 mm diameter glass capillary, and measurements were performed at room temperature with an irradiation time of 10 min.

Conversion from two-dimensional diffraction images to one-dimensional intensity profiles was carried out using 2DP software (Rigaku, Japan). The degree of crystallinity was determined based on the intensity of peaks obtained from the one-dimensional X-ray profiles using the following Eq. (1):

1 $${X}_{c}=\frac{{A}_{c}}{{A}_{c}{+A}_{a}}$$

where ${X}_{c}$, ${A}_{c}$, and ${A}_{a}$ represent the crystallinity, area of crystalline peaks, and area of amorphous peaks, respectively.

### Particle size distribution

To evaluate the particle size distribution of the prepared microbeads, optical microscope images were taken using an optical microscope (BX-53P, Olympus, Japan) equipped with a CCD camera (DP74; Olympus, Japan). The captured images were then analyzed using the image processing software ImageJ to derive the particle size distribution of various PHA microbeads. The size distribution histograms were plotted (Fig. S1).

### Compression testing of microbeads

Compression testing of microbeads was performed according to JIS Z8844:2019 standards on a micro compression tester (MCT-211; SHIMADZU, Japan), using a 50 μm flat-tip diamond indenter. The particle size of the microbeads was measured using an optical microscope mounted on the compression tester. Each microbead was subjected to a compression test force increasing from 0 to 200 mN at a rate of 4.8 mN/s. Since plastic particles are soft and do not exhibit a definite crush point, compressive strength was derived from the stress at 10% displacement. Microbeads from each polymer was tested using n = 7 distinct samples, and the average value was reported.

### Enzymatic degradation

Enzymatic degradation of all the PHA microbeads prepared in this study was carried out using an extracellular P(3HB) depolymerase from *Ralstonia pickettii* T1. Briefly, to each microbead (approximately 10 mg) placed in a microtube, 1 mL of 1 mol/L phosphate buffer (pH 7.4) was added, along with 8 mg (specific activity 7.8 U/mL) of P(3HB) depolymerase. The microbeads in the buffer were then incubated at 37 °C with shaking. After 1, 3, and 7 days from the start of the test, a portion of the microbeads was collected. The recovered microbeads were washed with distilled water and air-dried, and morphological observations were made using SEM.

### Deep-sea degradation tests of PHA microbeads

In situ marine degradation of PHA microbeads were tested at the bathyal seafloor off Misaki port in the northern Pacific Ocean (Table 2). Misaki port in the Sagami Bay is one of the most surveyed areas in the coastline and also considered to be a prime spot for plastic accumulation as it is close to highly populated areas^51,52^. In terms of sample preparation, microbeads were initially sieved and separated to particles with > 50 µm. These microbeads were then placed in a nylon bag with mesh size 50 µm and hot-sealed using a sealing machine. The mesh bags were then put into plastic bottles with several open holes to enable free flow of water and marine snow in and out of the bottles. The deployment and recovery of the samples were then carried out using a human occupied submersible *Shinkai6500* (on board its support vehicle RV *Yokosuka)*. After recovery, the samples were immediately stored in a freezer at − 80 °C until further characterization. A part of PHB4HB samples were immobilized with formaldehyde to study the biofilm morphology. All the recovered samples (except formaldehyde immobilized) were first ultrasonicated in distilled water for about a min to remove the sediment adhered to the surface of the microbeads, and then dried under vacuum overnight. The percentage recovery was calculated based on the initial and recovered weight of the beads. Biofilm attached sample was washed with pure water, dehydrated gradually using a series of ethanol washes, substituted with tert-butyl alcohol and finally freeze dried. The samples were then subjected to morphological analysis using the same protocol described earlier.

### Biochemical oxygen demand (BOD) tests

To quantitatively assess the biodegradability of PHA microbeads in the marine environment, a BOD study was conducted. Seawater and soil samples were collected from Tokyo Bay (35°37ʹ N, 139°46′ E) on September 8, 2022, with water temperature of 27 °C. BOD test water was prepared by mixing 5 L of seawater with 1 kg of soil, allowing the mixture to stand at room temperature for one week, and then filtering it.

BOD tests were performed using a temperature-controlled BOD measuring device (OxiTop; WTW, Germany) under aerobic conditions at 25 °C for 25 days with stirring. Approximately 6 mg of each PHA microbead or cellulose reference, along with 100 mL of test water, were added to each 300 mL BOD measuring device. Additionally, 0.1 mL of buffer, 25 mg of NH_4_Cl, 5 mg of NaHPO_4_, and 0.5 mg of allylthiourea were introduced to the test water. The buffer solution contained (per L): 33.3 g NaHPO_4_-2H_2_O, 21.8 g K_2_HPO_4_, 8.5 g KH_2_PO_4_, and 1.7 g NH_4_Cl.

BOD data were collected daily, and BOD biodegradability was calculated using the following Eq. (2):

2 $$\mathrm{BOD\, biodegradability}\left(\mathrm{\%}\right)=\frac{{{\text{BOD}}}_{{\text{t}}}-{{\text{BOD}}}_{{\text{b}}}}{{\text{ThOD}}}\times 100\mathrm{\%}$$

where BOD_t_, BOD_b_, and ThOD represent the BOD of the test solution, BOD of the control blank, and theoretical oxygen demand, respectively. Three samples were used for each BOD test, and the BOD biodegradability of each sample was determined as the average of the three results.

### Supplementary Information

Supplementary Figure S1.

## Data Availability

The datasets generated during the current study are available from the corresponding author on reasonable request.
