# Supplementary material for: Coastal and deep-sea biodegradation of polyhydroxyalkanoate microbeads
Source: Sci Rep. 2024 May 5;14:10302. doi: 10.1038/s41598-024-60949-z (PMC11070421; doi:10.1038/s41598-024-60949-z)
Supplement: Supplementary file 1 — Supplementary Figure S1. [file 41598_2024_60949_MOESM1_ESM.pdf]

Coastal and deep-sea biodegradation of polyhydroxyalkanoate microbeads

Natsumi Hyodo [1], Hongyi Gan [1], Manikandan Ilangovan [1], Satoshi Kimura [1], Ken-ichi Kasuya [2], Noriyuki Isobe [3] and Tadahisa Iwata [1,*]

[1] Science of Polymeric Materials, Department of Biomaterial Sciences, Graduate School of Agricultural and Life Sciences, The University of Tokyo, 1-1-1 Yayoi, Bunkyo-ku, Tokyo 113-8657, Japan

[2] Green Polymer Research Laboratory, Graduate School of Science and Technology, Gunma University, Kiryu, Gunma 376-8515, Japan

[3] Biogeochemistry Research Center, Research Institute for Marine Resources Utilization (MRU), Japan Agency for Marine-Earth Science and Technology (JAMSTEC), 2-15 Natsushima-cho, Yokosuka, Kanagawa 237-0061, Japan

[*] Corresponding Author: Tadahisa Iwata, E-mail address: atiwata@g.ecc.u-tokyo.ac.jp,

Telephone : +81 3-5841-5266 Fax: +81 3-5841-1304

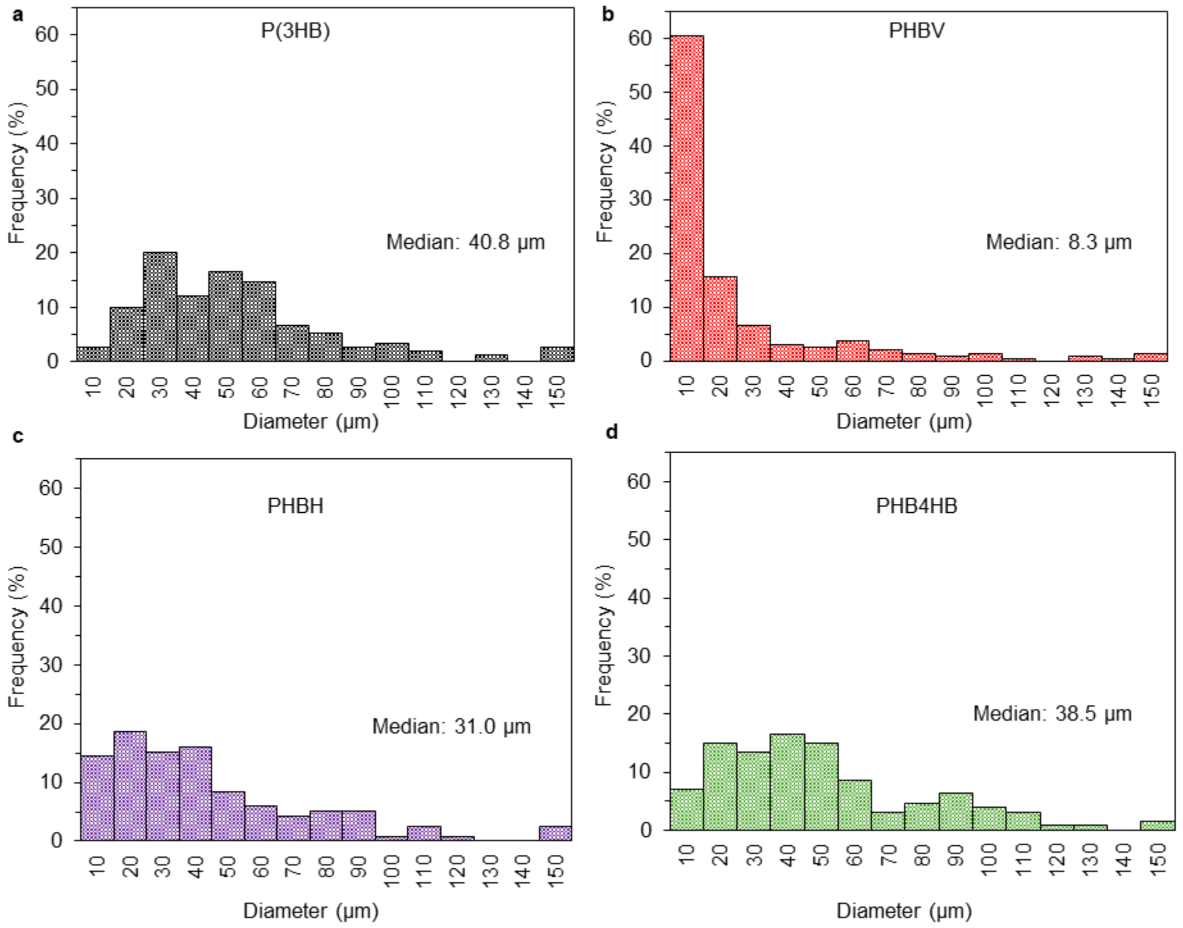

Fig. S1 The particle size distribution of the prepared PHA microbeads. The sample names and the median diameter are both indicated within the distribution graph.
